# Recurrent Facial Paralysis in a 14-Year-Old Due to Brazilian Lyme Disease: A Case Report

**DOI:** 10.7759/cureus.73843

**Published:** 2024-11-17

**Authors:** Carlos E Levischi Jr

**Affiliations:** 1 Internal Medicine, Hospital Israelita Albert Einstein, São Paulo, BRA

**Keywords:** facial nerve paralysis, lyme borreliosis, lyme disease, lyme disease and other tick-borne pathogens, neurological lyme

## Abstract

Lyme disease is a prevalent infection in the northern hemisphere, affecting approximately 450,000 new cases annually in the United States and 65,000 in Europe. This illness is spread by the bite of ticks harboring *Borrelia* spirochetes and develops in three progressive phases. In the second phase, neurological complications are common, including cranial nerve involvement. Notably, facial nerve neuropathy occurs in about 10% of adults with neurological symptoms, with an even higher incidence among pediatric cases.

In this context, a 14-year-old female patient presented with acute facial paralysis that initially improved but recurred on the same side several months later. Comprehensive evaluation led to a diagnosis of Lyme disease, and following targeted treatment, she experienced a marked recovery of her symptoms.

## Introduction

Lyme disease (LD) is a non-contagious systemic infection caused by a spirochete bacterium from the *Borrelia* genus [1]. First identified in 1975 in Old Lyme, USA, the disease initially presented as arthritis and erythema migrans, a distinctive skin lesion. Brazil saw its first cases documented in 1987 [2].

Transmission of *Borrelia* occurs through ticks, notably the *Ixodes ricinus* *complex* in the northern hemisphere [3]. In Brazil, the disease is believed to be transmitted primarily by *Amblyomma cajennense* (commonly known as the star tick), although other species such as *Rhipicephalus bovis* (cattle tick), *Dermacentor nitens* (horse tick), and *Rhipicephalus sanguineus* (dog tick) may also act as vectors and occasionally parasitize humans [4].

Despite its established presence globally and being particularly endemic to North America, Europe, and Asia, LD is rarely diagnosed in Brazil. However, cases have been documented across the country, indicating its wider reach [5]. The disease progresses through three distinct clinical stages, each potentially exhibiting different symptoms based on the affected tissues, immune response, and infection duration [4]. Notably, not all patients experience every stage, and symptoms may vary.

All manifestations of borreliosis warrant antibiotic treatment, with the choice and duration of antibiotics tailored to the disease's stage and severity. For instance, erythema migrans, cutaneous lymphocytoma, Lyme arthritis, and acrodermatitis chronica atrophicans respond well to oral antibiotics. However, severe neurological, cardiac, or ocular symptoms call for intravenous antibiotics initially, with doxycycline, amoxicillin, cefuroxime, or, if necessary, azithromycin available for oral treatment. For intravenous use, ceftriaxone, cefotaxime, and penicillin G are prescribed [6], while benzathine penicillin is an intramuscular alternative [7].

A thorough diagnostic evaluation is essential before beginning treatment, except for erythema migrans, to exclude other conditions with overlapping symptoms. Furthermore, serological testing is not recommended to monitor LD treatment efficacy due to persistent antibodies post-infection, complicating result interpretation in patients with previous LD history [8].

## Case presentation

A 14-year-old female patient, born and raised in Brazil, living in an urban area in the interior of the state of São Paulo, sought emergency medical care due to a loss of movement in the right hemifacial region. At the time of evaluation, a diagnosis of Bell's palsy, grade III, moderate dysfunction on the House and Brackmann scale, was suggested. Corticosteroid therapy was initiated, and physical therapy was recommended. The medication prescribed was prednisone, with an initial dose of 60 mg, followed by a gradual taper over a total treatment period of two weeks. There was a gradual reduction of symptoms, and within four weeks, the condition had completely resolved.

Two months after the initial episode, a new episode of facial paralysis occurred on the same side, with the same characteristics. At that time, corticosteroid therapy was restarted at the same dosage as before. Subsequently, the patient was evaluated by an infectious disease specialist, and several serological tests were performed, including for LD. Serology for herpes 1 and 2, cytomegalovirus, Epstein-Barr, rubella, and varicella-zoster revealed positive IgG and negative IgM results for all. Serology for toxoplasmosis was negative for both IgG and IgM. The venereal disease research laboratory (VDRL) test was negative, while LD serology was positive for IgM in both the enzyme-linked immunosorbent assay (ELISA) test and Western blot. A cranial magnetic resonance imaging (MRI) was also performed, with no abnormalities detected.

Given the patient's clinical presentation, a diagnosis of LD was made, and treatment was initiated. The chosen therapy was ceftriaxone, 2 g per day for 28 days. The regimen was administered via a peripherally inserted central catheter (PICC), with daily infusions performed by a homecare team. The treatment proceeded as planned without complications, and the facial paralysis fully resolved within two weeks. The patient was monitored throughout the treatment, with no abnormalities in liver or kidney function observed during or after the infusion period.

After four years of follow-up, there were no further episodes of facial paralysis or any other manifestations of LD.

## Discussion

The initial stage of LD, known as acute or localized disease, typically occurs 3-30 days post-tick bite, with erythema migrans appearing at the bite site. Nonspecific symptoms, such as fever, muscle aches, joint pain, headache, neck discomfort, sore throat, and lymph node enlargement, may also arise but often resolve on their own over time.

The second phase, termed the early disseminated stage, can emerge weeks or months after exposure. Symptoms may now involve multiple systems: the skin, heart, eyes, and nerves. Up to 15% of patients may show neurological symptoms, including meningitis, encephalitis, radiculoneuritis, and cranial nerve neuropathy.

In the third stage, also known as chronic or late disseminated disease, symptoms can appear months or years later, encompassing arthritis, cognitive decline, depression, and, in some cases, psychiatric disturbances [7]. In children, LD peaks between ages five and nine, predominantly affecting boys [9].

Diagnosing LD in the presence of erythema migrans is mainly clinical, as antibodies can take 4-6 weeks to be detectable in some cases. Thus, laboratory tests are generally unnecessary at this point [10]. For later stages, serological testing through a two-step method is recommended: an initial ELISA followed by a confirmatory Western blot for positive or indeterminate results [11]. In cases of suspected neuroborreliosis, clinical presentation combined with blood serology typically suffices, without necessitating cerebrospinal fluid analysis unless to exclude other conditions [12]. Importantly, cerebrospinal fluid interpretation for LD diagnosis lacks standardized criteria, unlike blood tests [13].

Cranial nerve neuropathy is one of the most prevalent neurological manifestations in cases of neuroborreliosis, with the seventh cranial nerve being predominantly affected. Although rare, other cranial nerves can also be implicated in the disease's progression [14]. In pediatric patients with neurological manifestations of LD, facial paralysis is observed in nearly 50% of cases [15,16]. Typically, facial paralysis presents as bilateral facial weakness, impacting both the upper and lower sections of the face. Bilateral facial paralysis is relatively common, with studies indicating a prevalence of 22-35% in such cases [17,18]. Recurrence of facial paralysis is also a notable characteristic of Lyme borreliosis, especially when treatment is inadequately administered [19]. Alongside facial paralysis, patients may experience symptoms like erythema migrans, general malaise, temporomandibular dysfunction, and joint pain.

Diagnosis of facial paralysis generally relies on clinical history and physical examination, with imaging studies often deemed unnecessary. However, imaging may be recommended in cases with atypical clinical presentations, slow or incomplete recovery, or recurrent episodes. The choice of imaging modality should be guided by clinical presentation and availability. Specifically, gadolinium-enhanced MRI is ideal for assessing lesions in the parotid gland, cerebellopontine angle, and internal auditory canal, whereas high-resolution computed tomography (CT) is more suited for evaluating temporal bone pathologies [20].

Antibiotic therapy generally does not alter the course of facial paralysis itself but is crucial for preventing long-term complications associated with LD. Corticosteroids are not recommended in pediatric cases of facial paralysis secondary to neuroborreliosis [8]. For patients with eyelid involvement, ophthalmologic management should include artificial tears and night-time eye patching. Additional therapies, such as physiotherapy, localized massage techniques, biofeedback, and acupuncture, may support quicker recovery in cases of facial paralysis [6]. The overall prognosis for facial paralysis in LD cases is favorable, with the majority of patients achieving complete recovery [8].

## Conclusions

LD presents a clinical complexity and is relatively uncommon in Brazil, yet it should be considered a differential diagnosis in cases of bilateral or recurrent facial paralysis, including pediatric patients. Importantly, the disease can reach disseminated stages without preceding symptoms from earlier phases or a clear history of tick exposure. This underscores the need for emergency physicians, pediatricians, and otolaryngologists to remain vigilant for LD in patients presenting with facial paralysis, even in the absence of typical initial symptoms or documented tick bites.
